# Recommendations and Nutritional Considerations for Female Athletes: Health and Performance

**DOI:** 10.1007/s40279-021-01508-8

**Published:** 2021-09-13

**Authors:** Bryan Holtzman, Kathryn E. Ackerman

**Affiliations:** 1 Perelman School of Medicine, University of Pennsylvania, Philadelphia, PA USA; 2 Female Athlete Program, Division of Sports Medicine, Boston Children’s Hospital, Boston, MA USA; 3 Neuroendocrine Unit, Massachusetts General Hospital, Boston, MA USA; 4 Harvard Medical School, Boston, MA USA

## Abstract

Optimal nutrition is an important aspect of an athlete’s preparation to achieve optimal health and performance. While general concepts about micro- and macronutrients and timing of food and fluids are addressed in sports science, rarely are the specific effects of women’s physiology on energy and fluid needs highly considered in research or clinical practice. Women differ from men not only in size, but in body composition and hormonal milieu, and also differ from one another. Their monthly hormonal cycles, with fluctuations in estrogen and progesterone, have varying effects on metabolism and fluid retention. Such cycles can change from month to month, can be suppressed with exogenous hormones, and may even be manipulated to capitalize on ideal timing for performance. But before such physiology can be manipulated, its relationship with nutrition and performance must be understood. This review will address general concepts regarding substrate metabolism in women versus men, common menstrual patterns of female athletes, nutrient and hydration needs during different phases of the menstrual cycle, and health and performance issues related to menstrual cycle disruption. We will discuss up-to-date recommendations for fueling female athletes, describe areas that require further exploration, and address methodological considerations to inform future work in this important area.

## Key Points

**Table:** 

Female athletes should aim for energy availability (EA) of 45 kcal·kg^–1^ fat-free mass·day^–1^ for optimal health and performance; optimizing nutrient composition based on menstrual cycle phase is ineffective without the requisite energy for basic functioning.
Micronutrient deficiencies are common in female athletes, particularly in iron, vitamin D, and calcium; nutritional strategies should be used to prevent these deficiencies, including increasing consumption of diverse foods and potential supplementation.
Micro- and macronutrient requirements, as well as hydration needs, may change during various phases of the menstrual cycle as a result of hormonal fluctuations.

## Introduction

Female athletes make up nearly 50% of sports participants. Unfortunately, research into optimizing nutrition for health and performance specific to female physiology is lacking. In this review, we will describe the challenges of studying women, the potential pitfalls of applying research from males to females, provide recommendations for adequate caloric intake, describe sequelae of insufficient caloric intake, propose a simple framework for designing nutrition plans for female athletes, and outline basic recommendations for nutrition plans for female athletes with resources for further reading.

## Research in (Wo)men

As is the case in many medical fields, sports science research has a paucity of female-specific inquiries, leading to the misapplication of findings from male subjects to female athletes. In 2011–2013, studies published in three of the world’s top sports medicine journals (*British Journal of Sports Medicine*, *Medicine and Science in Sports and Exercise*, and *American Journal of Sports Medicine*) had women representing 39% of study participants and only 4% of studies were female-only [1, 2]. Follow-on studies in the latter two journals showed continued trends through the first half of 2015; for example, for studies of athletic performance, 63% were conducted in male subjects only, 33% in male and females, and a paltry 3% were solely focused on female athletes [2].

**Fig. 1:**
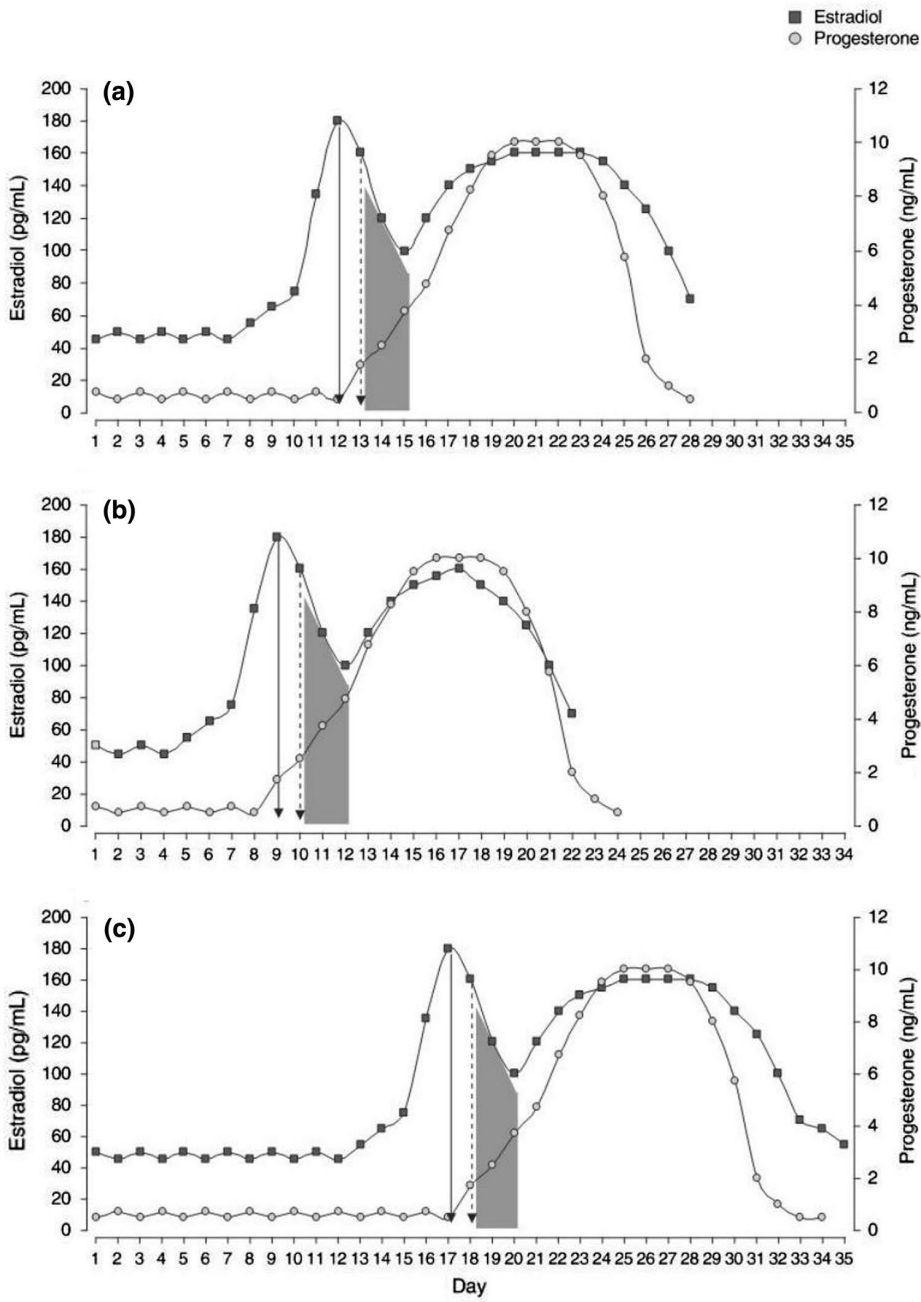
Hypothetical examples of the hormonal profiles of three eumenorrheic women with different cycle lengths. **a** 28-day cycle; **b** 22-day cycle; **c** 35-day cycle. Solid arrow indicates estradiol peak; dashed arrow indicates luteinizing hormone peak; shaded area indicates ovulation. Reproduced from Vescovi with permission [3]

Women are often cast aside as being “more difficult” to study than men due to their higher level of hormonal intricacy. No two menstrual cycles are the same, both for one woman cycle to cycle and when comparing women. Some aspects related to the hypothalamic–pituitary–gonadal (HPG) axis that must be considered when studying female athletes are listed in Table 1. Eumenorrhea is typically defined as regular menstrual cycles that last 21–35 days; Fig. 1 illustrates the different hormonal status of three “normal” menstrual cycles [3]. Because of their pulsatile release patterns, proper characterization of luteinizing hormone (LH) and follicle-stimulating hormone (FSH) levels requires overnight blood sampling at minimum 3x/hr and preferably 6x/hr, further adding to research costs and invasiveness [4]. These hormonal factors necessitate studies of female athletes to have a larger number of groups within each study: for example, stratification by menstrual status, by contraceptive use (including type, formulation), and pregnancy status. This all adds to the complexity and cost of well-designed studies.

**Table 1:** Hypothalamic–pituitary–gonadal axis considerations when designing studies in female athletes

**Menstrual cycle**	Phase
	Oligo-amenorrhea
	Subclinical menstrual defects
	Cycle length
	Number of bleeding days
	Amount of bleeding
	Polycystic ovary syndrome
**Gynecologic history**	Gravidity
	Parity
	Age of menarche
	Age of menopause
**Hormonal contraception**	Hormone(s) used
	Composition of hormone
	Route of administration
	Length of use
**Anatomy**	Congenital defects
	Surgical status
**Disease history**	Malignancy

The only female-centric component of exercise physiology and sports medicine to receive considerable research attention is the female athlete triad (the triad). First described by Barbara Drinkwater and colleagues in 1984 [5] and named in 1993 [6], research surrounding the triad and its associated negative sequelae has put these parts of female athlete health in the spotlight [7]. Fewer studies, however, have addressed female athlete performance and female-specific physiology for athletes. This has led to potentially incorrect application of findings in male athletes to female athletes.

## Nutrition Gone Wrong: Relative Energy Deficiency in Sport (RED-S)

A considerable proportion of female athletes will experience nutritional deficiencies at some point in their careers. These deficiencies can arise through a variety of pathologies, including inadvertent undereating, knowledge deficit, food insecurity, time constraints, restrictive dietary habits, and frank eating disorders (EDs) such as anorexia nervosa [8]. Traditionally, athletes who compete in endurance sports, aesthetic sports, or weight class sports are thought to be most likely to have inadequate nutrition for their training or competition, either due to a perceived performance benefit or requirement for competition [9–11]. Estimates of ED or disordered eating (DE)—typically low energy availability (EA) states—prevalence among female athletes are variable at 6–45% [12]. A recent survey of 1000 female athletes age 15–30 years across ≥ 40 sports estimated the risk of low EA at 47.3% [13].

Quantification of energy status can be difficult outside the lab [14]. EA is the recommended measure of nutritional energy status for athletes [15] and is calculated as energy intake (EI) minus exercise energy expenditure (EEE) normalized to fat free mass (FFM) per day: ${\text{EA}} = \frac{{{\text{EI~}}\left( {{\text{kcal}}} \right) - {\text{EEE~}}\left( {{\text{kcal}}} \right)}}{{{\text{FFM~}}\left( {{\text{kg}}} \right)}}{\text{~day}}^{{ - 1}}$ [16, 17]. Put plainly, EA quantifies the amount of caloric energy that can be used by the body for physiologic functioning (e.g., homeostasis, thermoregulation, anabolism) after accounting for the energy used for training.

Despite the difficulties of quantifying EA for free living athletes and using it to guide nutritional strategies, a substantial body of research has used the concept to define the levels of energy deficit when different physiologic changes occur. Early work suggested that EA < 30 kcal kg^−1^ FFM·day^−1^ disrupted LH pulsatility, and this level became the benchmark at which “low EA” was defined [18]. EA of approximately 45 kcal kg^−1^ FFM·day^−1^ may be the ideal level for maintenance of body mass and allow for athletes to focus on skill development while EA > 45 kcal kg^−1^ FFM·day^−1^ provides enough energy for weight gain and muscle hypertrophy [19]. Considerable investigation has focused on defining an EA threshold with clinical meaning. It is probable that no universal binary threshold exists. One study of college-aged eumenorrheic sedentary women exposed to different EAs through manipulation of diet and exercise found a linear relationship between EA and the incidence of menstrual disturbances [20]. EA of 30 kcal kg^−1^ FFM·day^−1^, however, has been shown to discriminate between amenorrheic vs. eumenorrheic status while not distinguishing the presence of subclinical disturbances [21]. A long-term, periodized approach to changing EA and body composition based on stage in the competitive season may permit overall sufficient EA with low risk for negative sequelae with short periods of improved power-to-weight ratio [22]. Further investigation into the EAs at which different physiologic disturbances occur is warranted.

### Female Athlete Triad: EA, Bone Health, and Menstrual Function

**Fig. 2:**
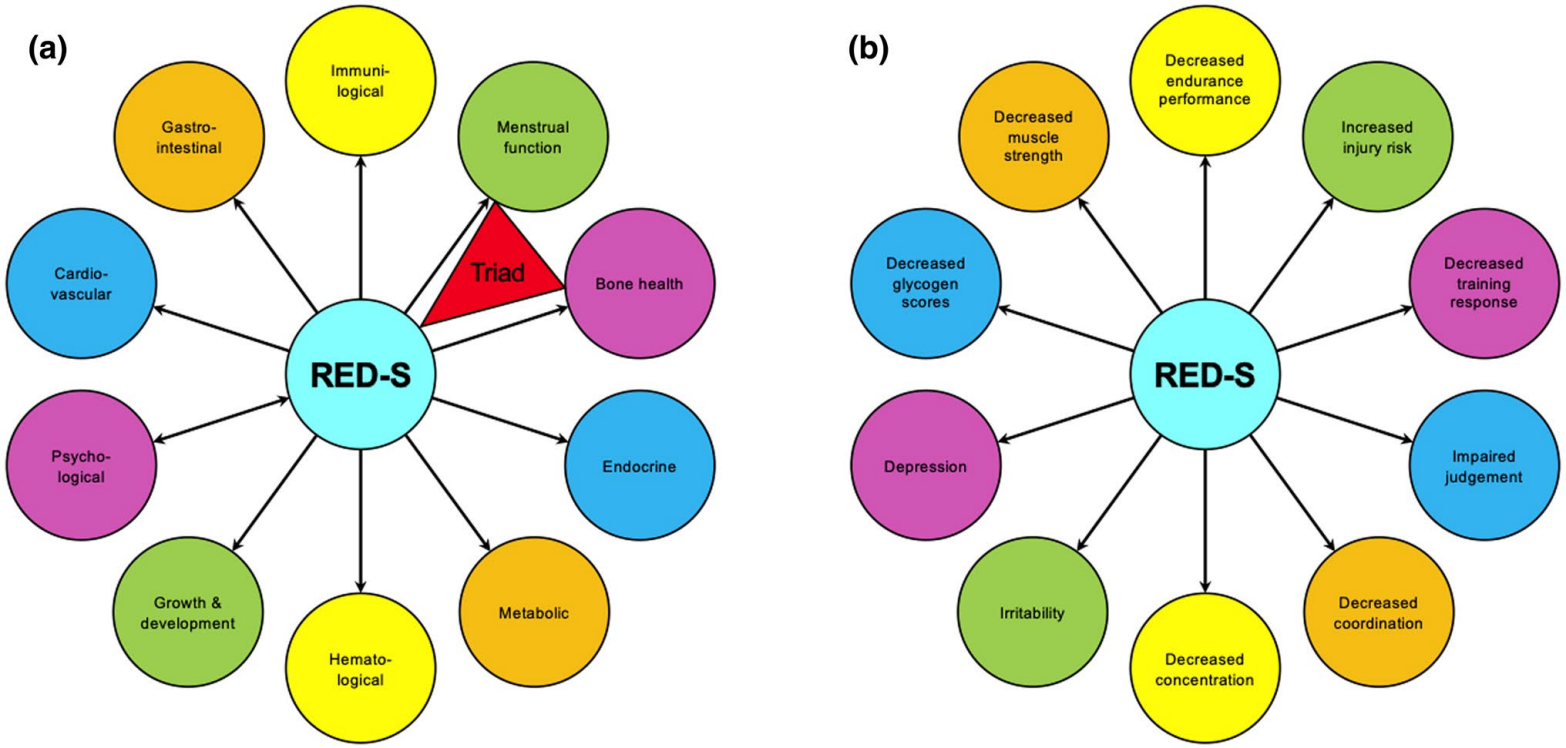
**a** Health consequences of relative energy deficiency in sport (RED-S); **b** performance effects of RED-S. Adapted from Constantini (with permission) [25]

RED-S is a comprehensive model that outlines some of the health and performance consequences for athletes with low EA [23–25] (Fig. 2). The well-established female athlete triad [26]—the syndrome of perturbations of EA, bone health, or menstrual status—is included as a subset of RED-S. Each component of the triad can arise independently and in isolation [16], however, low EA can account for part of the pathology in menstrual dysfunction and bone health via disruption of the HPG axis. As mentioned, low EA causes disruptions of LH pulsatility, which is used as a surrogate for hypothalamic gonadotropin-releasing hormone (GnRH) pulsatility (GnRH is not systemically released and direct sampling requires transsphenoidal cannulization of the portal hypophyseal system). Disruptions to GnRH pulsatility disturb gonadotropin release, which in turn causes menstrual dysfunction (manifest as oligo-amenorrhea) [27]. Irregular or absent menses, in concert with abnormal pituitary signaling, causes systemic reductions in estradiol [28]. Estradiol is an osteoprotective agent that inhibits osteoclast activity, ultimately promoting more bone formation than resorption. When estrogen levels are aberrantly subphysiologic, osteoclast activity predominates and bone mass is lost. While this model depicts low bone mineral density (BMD) as the endpoint of low EA, it is important to consider that undernutrition can directly cause low BMD as well [29]. A study of eumenorrheic young women exposed to 5 days of reduced EA (10, 20, or 30 kcal kg^−1^ FFM·day^−1^, achieved through manipulation of EI with constant EEE of 15 kcal kg^−1^ FFM·day^−1^) demonstrated a dose–response relationship between EA and bone remodeling markers while maintaining normal menstrual function [30]. However, this study did not investigate the long-term effects of energy suppression on bone function and the subjects experienced decreased levels of estradiol, which may have led to menstrual dysfunction with extended study duration [30]. Furthermore, in the setting of oligo-amenorrhea, bone microarchitecture is negatively affected. While normal athletic activity increases tibial cross sectional area, oligo-amenorrhea decreases trabecular number and cortical thickness, leading to decreases in trabecular and total BMD, ultimately resulting in decreased stiffness and lower failure load [31–33]. These changes all lead to increased incidence of bone stress injury.

### Health Consequences of RED-S

The best-studied components of RED-S are those included in the triad model. The body of evidence for the other components, however, continues to grow. Due to their integral role in exercise physiology, the endocrine alterations that occur in low energy states have been extensively reviewed [27]. After accounting for methodological differences, low EA seems to cause the following hormonal alterations in exercising women: decreased estradiol, decreased progesterone, decreased leptin, increased ghrelin, increased adiponectin, increased peptide YY (PYY), decreased insulin, increased cortisol, decreased total triiodothyronine (T3) and free T3, decreased free thyroxine (T4), increased growth hormone (GH) (with increased growth hormone resistance), and decreased insulin-like growth factor 1 (IGF-1) [27]. GH/IGF-1 dysregulation may play a role in stunted linear growth [34]. How changes in each hormone, both in isolation and in concert with other changes, affects athletic health and performance remains to be fully elucidated.

The most common hematological change experienced by energy deficient athletes is iron deficiency. At baseline, 24–47% of women experience iron deficiency without anemia [35]. Mechanisms of iron deficiency in the setting of energy deficiency include inadequate dietary intake, impaired absorption, and foot strike hemolysis (exacerbated by aberrant exercise habits) [36]. Iron deficiency can worsen the hypometabolic state that accompanies low EA by impairing T4 synthesis [37] and hepatic conversion of T4 to the active metabolite T3 [38]. Compounding the problem, iron deficiency can promote energy deficiency by shifting ATP production away from oxidative phosphorylation to less efficient anaerobic pathways and reducing the expression of iron-dependent enzymes while upregulating the expression of iron-independent enzymes involved in metabolism [39]. Iron deficiency can also contribute to poor bone health in RED-S through suppression of the osteotrophic hormones growth hormone and IGF-1, changes in bone microarchitecture, and the aforementioned changes to the hypothalamic–pituitary–thyroid axis [39].

Perhaps paradoxically, negative cardiovascular changes can occur in energy deficient athletes. In severely energy deficient athletes, symptomatic bradycardia, arrythmias, and frank hypotension can present and are criteria for hospitalization [40]. In women, hypoestrogenism experienced in oligo-amenorrhea induces a post-menopausal-like physiology: endothelial dysfunction [41, 42], poor lipid profiles (low-density lipoprotein (LDL), total cholesterol, triglycerides) [41, 43, 44], and renin–angiotensin–aldosterone axis changes have all been observed [45]. The long-term effects of the functional hypothalamic amenorrheic hypoestrogenic state on vascular health has not been investigated.

Immunologic dysfunction has recently been questioned as an effect of low EA [46]. Traditional thinking dictated that innate and acquired immunity were temporarily lowered during periods of heavy training [46]. It is likely, however, that athletes are more frequently subjected to standard risk factors for illness—e.g., psychological stress [47], poor sleep [48], and travel [49, 50]. In terms of energy deficiency, observational data in athletes and controlled data from anorexia nervosa studies are at odds. Recent survey data from the 2016 Rio Olympics showed an association between low EA (assessed by the Low Energy Availability in Females Questionnaire (LEAF-Q) [51]) and infectious symptoms [52, 53]. Anorexia nervosa, however, seems to confer protection against infections in milder forms of disease (body mass index (BMI) > 15 kg m^−2^)—reflective of the severity of energy deficiency in most training and competing female athletes [54–56]. Preservation of protein intake [57], allowing for adequate immune synthesis, may play a role in maintaining immunity despite energy deficiency [58–60]. Further understanding of immune mechanisms with low EA is warranted.

Likely an evolutionary response to food scarcity to conserve calories, resting metabolic rate (RMR) decreases in energy deficiency. A study of 40 female weight-bearing endurance athletes with optimal EA (≥ 45 kcal kg^−1^ FFM·day^−1^), reduced EA (30–45 kcal kg^−1^ FFM·day^−1^) and low EA (< 30 kcal kg^−1^ FFM·day^−1^) found that the optimal EA group had a significantly higher RMR than the reduced/low group [61]. In addition, eumenorrheic athletes had higher RMR than athletes with menstrual dysfunction [61].

Gastrointestinal changes for athletes in the setting of low EA remain poorly defined; the only published studies are cross-sectional or questionnaire-based. A 1000 athlete study found increased rates of fecal incontinence and constipation in those at risk for low EA [13] while the original LEAF-Q study showed athletes with low EA had greater odds of experiencing gastrointestinal symptoms (≥ 2 of bloating, cramps, ≠ 1 bowel movement/day, regular diarrhea or hard stools) [62]. A recent small study of elite athletes using the LEAF-Q found no evidence of gastrointestinal dysfunction in those with low EA, though it is probable this study was underpowered [63]. New evidence regarding urinary incontinence in female athletes with low EA may provide further causal mechanisms for fecal incontinence through shared pelvic floor dysfunction [64].

In the original RED-S model, psychological illness is described as a cause and effect of low EA [23]. A full discussion of the spectrum of mental health disorders experienced by female athletes is beyond the scope of this review; however, the importance of athletes’ mental health has recently captured the attention of popular media and international sporting organizations [65]. Treatment of underlying psychiatric comorbidities is critical in treating energy deficiency. Insight from anorexia nervosa treatment has shown that nutritional repletion is necessary for improving mood [66], anxiety [66], and cognitive function [67, 68], and clinical experience has shown that patients’ insights into their illnesses—and thus success of treatment—improves with proper nourishment. As such, we recommend focusing on early nutritional replenishment when treating psychiatric disease in the setting of low EA.

### Performance Effects of Low EA

The RED-S model proposed 10 negative performance detriments that occur due to low EA: increased injury risk, decreased training response, impaired judgment, decreased coordination, decreased concentration, increased irritability, higher rates of depression, decreased glycogen stores, decreased muscle strength, and decreased endurance performance [23]. Interventional studies on these performance effects are sparse because of absent athlete buy-in for a study hypothesized to worsen competitiveness and difficulties with ethical approval. Furthermore, some of the performance effects are vague, and how those detriments manifest in the competitive arena is difficult to assess. Nevertheless, one study monitored 10 adolescent female swimmers over a competitive season (12 weeks) and compared 400 m freestyle times between eumenorrheic and ovarian suppressed athletes [69]. The suppressed group had lower estradiol and progesterone levels throughout the season and, at the end of the study, had decreased EI, EA, T3, and IGF-1 compared to the eumenorrheic group [69]. At the end of the season, the ovarian suppressed group had a 9.8% increase in time (i.e., worse performance) and the eumenorrheic group had an 8.2% improvement [69]. Another study of elite rowers over 4 weeks of energy deficiency induced by increased training load found decrements in 5 km time, boat velocity, and stroke rate [70]. This study may be limited because it did not allow adequate time for supercompensation following an intensified training block. These rowers also experienced increased fatigue, more sleep disturbances, and greater total mood disturbance throughout the training block and increasing energy debt [70].

## Nutritional Recommendations for Female Athletes

### Establishing the Hierarchy of Nutritional Needs

**Fig. 3:**
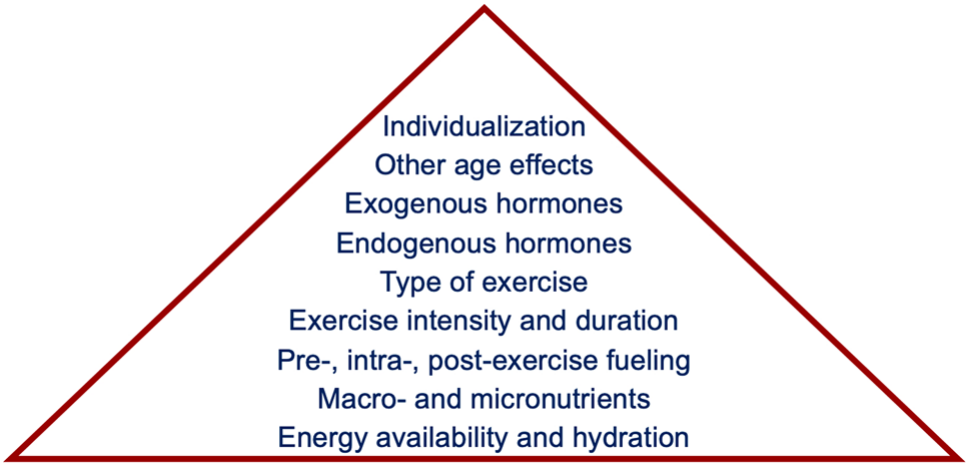
Potential hierarchy of nutritional considerations and needs for female athletes. When designing nutrition plans, athletes should ensure that all lower components are achieved when stepping up the pyramid

While many sources focus on certain isolated components of nutrition recommendations for female athletes, a holistic view of the female athlete needs to be used when designing a fueling plan. Our proposed model is shown in Fig. 3. At the base of our model is sufficient EA and hydration: for example, optimizing nutrient composition based on menstrual cycle phase is futile without the requisite energy for basic functioning. Once caloric needs have been considered, the composition of those calories—including both the three basic macronutrients (carbohydrate, protein, fat) and various micronutrients (vitamins and minerals)—can be tailored to the athlete’s need. Timing of nutritional intake, both throughout the day and before, during, and after exercise can then be optimized. Next, the length of exercise and the intensity of exercise, followed by the type of exercise, influences the athlete’s needs. For females, the endogenous cyclic hormonal profile can be input into the nutrition plan, followed by any exogenous hormone use. Then, any other age effects not already part of the above considerations can be altered, and, finally, individualization of the plan to the specific athlete. Both when designing guidelines for athletes in general and when working with specific athletes, this model can serve as a framework for the plans that are established. As the nutrition plan ascends the hierarchy, it becomes more prescriptive. It is important to remember that the best nutrition plan is the one that endows the best adherence. Similar to how athletes become more advanced in their training plans as training age and experience increase, nutrition plans should become more specific as the athlete becomes more experienced. Failure to proceed in a graduated fashion can overwhelm the athlete and hinder adherence the same way that too advanced a training plan can lead to injury.

Estrogen is the quintessential female hormone. Peak estrogen levels are reached on day 12–14 of a normal menstrual cycle; the high levels of estrogen agonize the hypothalamus to cause GnRH secretion, leading to massive LH secretion and induction of ovulation. During the subsequent luteal phase, estrogen levels again rise, and when they recede, menstruation occurs. Estrogen has pervasive anabolic effects, including improving muscle strength and BMD [71, 72]. During exercise, estrogen causes a protein-sparing effect: at intensities of approximately 65% maximal oxygen consumption (VO_2_max), women have higher rates of lipid oxidation and lower rates of carbohydrate and protein metabolism compared to men [73–75]. Estrogen also impairs gluconeogenesis. In the luteal phase, when estrogen levels are high for several days, female athletes are less reliant on muscle glycogen for fueling compared to exercise during the follicular phase and compared to male athletes [74, 76]. Increasing exogenous carbohydrate intake can overcome this impaired gluconeogenesis [74, 76].

### Nutritional Strategies for Endurance Athletes

#### Protein

Endurance sports have garnered the most research interest for female athletes. As previously mentioned, an EA target of approximately 45 kcal kg^−1^ FFM·day^−1^ is likely ideal for endurance athletes to maintain body mass and fuel high levels of training with fitness and performance improvements [19]. Recent investigation into the protein requirements of women exercising for 1.5 h/day showed that protein intake should be at least 1.6 g kg^−1^ day^−1^ during the follicular phase [77]. Because of increased progesterone levels in the luteal phase, the protein requirement may be higher due to higher rates of protein catabolism [78–81]. Currently, the American College of Sports Medicine (ACSM) recommends 1.2–2 g kg^−1^ day^−1^ protein intake distributed evenly throughout the day and after exercise [15]. It has been suggested that when designing the day-to-day diet, fulfilling this protein requirement should be the primary focus [82]. Athletes with vegan or vegetarian diets may struggle to meet this protein requirement without guidance from a sports dietitian.

#### Carbohydrate

Carbohydrate is the macronutrient that has received the most attention before, during, and after exercise. Carbohydrate availability is a limiting factor in performance of prolonged exercise [15]. Reduced carbohydrate availability is detrimental to exercise performance in two ways: (1) reduced muscle glycogen leads to fatigue and a drop in intensity, and (2) reduced circulating carbohydrate (i.e., blood glucose) for central neural nourishment impairs cognition [83, 84]. Importantly, glucose metabolized from muscle glycogen does not enter the systemic circulation; consequently, muscle carbohydrate availability can be adequate while systemic carbohydrate availability is low. These two components thus dictate carbohydrate intake strategies.

Gluconeogenesis rates are higher in the follicular phase than the luteal phase at exercise intensities > 50% *V*O_2_max [85, 86]. As such, a theoretical performance detriment could occur during the luteal phase due to impaired metabolism. Consuming a high carbohydrate snack 3–4 h before exercise can mitigate these effects during the luteal phase [82]. During exercise, current recommendations dictate consuming 30–60 g/h carbohydrate for durations 1–2.5 h and perhaps > 90 g/h carbohydrate for exercise durations > 2.5 h [15]; however, these recommendations are largely based on studies of male athletes [82]. Athletes should be wary of gastrointestinal upset caused by consuming fuel during exercise and overwhelming the gut; consequently, “gut training” should be incorporated into daily training and athletes should be reminded to avoid trying new strategies on competition days [87].

The existence of a post-exercise “window” for nutrient intake has been the subject of much interest in the popular media, and the understanding of the length of this window and optimal nutrient intake during it are shrouded by intrigue in the public mind. The goal of carbohydrate replenishment after exercise is to restore lost muscle and hepatic glycogen. Different strategies for this replenishment have been shown to be effective; current recommendations are for ≥ 1.2 g kg^−1^ h^−1^ carbohydrate intake for 4–6 h following the conclusion of a glycogen-depleting exercise session [88]. These recommendations, however, are based on studies of small numbers of male athletes and may not account for the differences in body composition in males and females. If this goal cannot be reached, adding protein in a 4:1 carbohydrate:protein ratio may aid in recovery and will not harm glycogen repletion [88]. The time between training/performance requirements will also dictate replenishment strategies (e.g., after a morning training session with an upcoming afternoon session vs. 90 min between events in a competition).

Carbohydrate loading—colloquially, “carboloading”—is another nutritional strategy that has long been in the public eye for improving endurance performance. The idyllic team “pasta party” the night before high school competitions is purported to provide the fuel for victory the next day. Carbohydrate needs are dependent on the duration and intensity of the competitive event. When interpreting published studies, it is important to remember that women oxidize more fat and less carbohydrate than men at the same relative intensities of exercise [89]. Metabolic clearance can be a significant limiting factor in exercise lasting under 90 min. Consequently, ensuring that glycogen stores are fully repleted is sufficient for maximizing performance, and this can be achieved with habitual intakes of 7–10 g kg^−1^ day^−1^ carbohydrate [90]. For events > 90 min, a dedicated 36–48 h period of high carbohydrate intake of 10–12 g kg^−1^ day^−1^ can improve performance when compared to no dedicated plan [91] and is the current recommendation for pre-competition carbohydrate loading [15, 90]. It is worth noting that these guidelines may be different in Paralympians, who have not been extensively studied, due to their lower muscle mass. Dedicated carbohydrate loading of 8.4–9 g kg^−1^ day^−1^ during the mid-follicular phase has shown improvements in muscle glycogen of 17–31% but no improvement in performance [92–94]. Carbohydrate loading in the mid-luteal phase, however, has shown no change [94] or small (13%) increase [95] in muscle glycogen with a potential for improved performance [95]. Oral contraceptive use further muddles the carbohydrate loading picture due to non-physiologic hormone levels and synthetic hormone use. One study investigated carbohydrate loading with women using a common ethinyl-estradiol/levonorgestrel formulation and found that muscle glycogen concentrations were similarly elevated in the purported mid-follicular and mid-luteal phases [96]; other formulations have not been investigated [82]. Finally, for a female athlete consuming a relatively low-calorie diet (e.g., 2000 kcal day^−1^), carbohydrate loading can be challenging: for a 55 kg athlete, consuming 8 g kg^−1^ day^−1^ carbohydrate would be 88% of her daily caloric intake. In light of these data, it is likely that the biggest benefit of the one-time pasta party the night before competition is for team bonding and relaxation prior to competition.

#### Fat

The role of fats in the diets of all athletes has long been debated and the high carbohydrate/low fat vs. low carbohydrate/high fat debate is outside the scope of this paper; readers are directed to a recent review by Louise Burke [97]. Athletes are encouraged to consume at least 20% of their calories in the form of fats [15]; failure to do so can lead to deficiencies in essential fatty acids and fat soluble vitamins (vitamins A, D, E, and K) as well as frank caloric insufficiency [98]. Contrary to popular opinion, increasing fat intake has not been shown to lead to increased adiposity in athletes [99, 100]. Furthermore, decreased fat intake has been correlated with higher rates of injury in female runners [101]. Omega-3 fatty acids are a commonly supplemented fat in the general public and may have a role in athlete health and performance. Omega-3 fatty acids may be protective against bone catabolism [102–104]. Currently, there are no specific recommendations for female athletes for omega-3 fatty acid intake and the Institute of Medicine recommends 1.2 g day^−1^ for women [105]. In the absence of specific recommendations for fat intake and composition, we recommend that female athletes consume at least 20% of their calories of fats from diverse sources to ensure repletion from a variety of fatty acids.

#### Micronutrients

Once primary macronutrient needs have been addressed, optimizing micronutrients can occur. In the setting of low EA, micronutrient deficiencies can occur due to overall inadequate nutritional intake. Deficiencies in iron, vitamin D, and calcium are common in female athletes; nutritional strategies should be adapted to prevent these deficiencies.

#### Iron

Estimates of iron deficiency prevalence vary from 15 to 35% [106–108] of female athletes to some studies suggesting rates > 50% [36, 109, 110]. Non-medical stakeholders, including the athlete herself, coaches, and parents, often misunderstand the interpretation of iron studies or want a single laboratory marker of iron deficiency. Often, ferritin is the first test in evaluation of iron deficiency. Ferritin is an iron-storing protein; however, it is also an acute-phase reactant, and values can be falsely elevated in the setting of illness or stress, masking a diagnosis of iron deficiency. A full iron panel includes a complete blood count with reticulocyte count (of interest: hemoglobin, hematocrit, mean corpuscular volume, mean corpuscular hemoglobin, mean corpuscular hemoglobin concentration, red blood cell distribution width), ferritin, serum iron, transferrin, transferrin saturation, and total iron binding capacity. “Iron deficiency” and “anemia” are often used interchangeably; however, they are not synonymous. Anemia is defined as a decreased red blood cell mass or hemoglobin concentration. The World Health Organization defines anemia based on hemoglobin concentrations: for women ≥ 15 years old, mild anemia is a hemoglobin 11.0–11.9 ng dL^−1^, moderate anemia is a hemoglobin 8.0–10.9 ng dL^−1^, and severe anemia is a hemoglobin < 8.0 ng dL^−1^ [111]. Proposed guidelines for definitions of iron deficiency severity for athletes are shown in Table 2 [112]. Thus, it is possible for an athlete to be iron deficient but not anemic; this syndrome is known as “iron deficiency” or “iron deficiency without anemia”. Athletes who are both iron deficient and anemic have the syndrome of “iron deficiency anemia”.

**Table 2:** Proposed guidelines by Peeling and colleagues for iron deficiency severity in athletes [112]

**Stage 1: Iron deficiency**	Iron stores in bone marrow, liver, spleen depleted Ferritin < 35 ng mL^−1^ Hemoglobin > 11.5 ng dL^−1^ Transferrin saturation > 16%
**Stage 2: Iron-deficient non-anemia**	Erythropoiesis diminishes as the iron supply to the erythroid marrow is reduced Ferritin < 20 ng mL^−1^ Hemoglobin > 11.5 ng dL^−1^ Transferrin saturation < 16%
**Stage 3: Iron-deficient anemia**	Hemoglobin production falls, resulting in anemia Ferritin < 12 ng mL^−1^ Hemoglobin < 11.5 ng dL^−1^ Transferrin saturation < 16%

Some female athletes are at inherently higher risk than others for iron deficiency. These athletes include those with restrictive diets (e.g., in order of increasing risk: no red meat, vegetarian, vegan), those with high amounts of repetitive ground strikes (e.g., sports involving high amounts of running) [113], endurance training causing antioxidant depletion and erythrocyte damage [114], and those with heavy menstrual bleeding [115]. Athletes can also have “pseudoanemia” where iron studies indicate anemia but the laboratory values are a result of expanded plasma volume [116]. The most common presenting symptom of iron deficiency in female athletes is poor athletic performance [117]. Iron from meat (heme iron) is better absorbed than plant-based iron (non-heme) [118], and meat iron sources have other nutrients that enhance heme iron absorption while plant iron sources have other nutrients that reduce non-heme iron absorption [119]. Athletes with restrictive diets that make adequate consumption of iron difficult should consult a sports dietitian to optimize their daily nutrition plans for iron intake. The current USDA recommended daily allowance for girls 14–18 years old is 15 mg day^−1^ and for women 19–50 years old is 18 mg day^−1^ [120]; however, athletes with the aforementioned risk factors should consumer higher levels of iron on a daily basis [15]. The US Armed Forces recommends that female soldiers—a highly active group of women—consume at least 22 mg day^−1^ iron [121].

Treatment of iron deficiency requires evaluation by a physician and sports dietitian to elucidate the cause of the deficiency and rule out any underlying pathophysiology. Empiric iron supplementation initiated by a non-medical professional could lead to continued ignorance of severe disease. Treatment strategies of iron deficiency depend on the severity of disease. As is the case with most diseases, conservative therapy is the first step; in the case of iron deficiency, this step is to increase dietary intake. Should increased dietary intake fail, oral supplementation of iron can be initiated, either in the form of liquid iron or iron tablets. A typical oral supplementation regimen is approximately 100 mg day^−1^ iron in divided doses for 8–12 weeks with supplementation with iron pro-absorptives, such as vitamin C [36, 122]. Slow-release ferrous sulfate formulations are recommended as the most effective and tolerable supplement [123]. Finally, intravenous iron supplementation is preferred to intramuscular injection for parenteral replenishment, and is reserved for special cases (e.g., severe deficiency, enteral absorption disruption) [36]. Readers are referred to a recent in-depth review by McCormick and colleagues for more information on treatment of iron deficiency in athletes [124].

#### Calcium and Vitamin D

Calcium levels are difficult to measure in a clinically meaningful matter for athletes because of the massive stores of calcium in bone and high sensitivity of the parathyroid to perturbations in calcium concentration. The best way to assess sufficient calcium intake is through retrospective dietary recall. Athletes at risk for low calcium should consume 1500 mg day^−1^ to optimize bone health [23]. Failure to do so can reduce BMD because of continued osteoclast activity from parathyroid hormone (PTH) stimulation occurring in response to low serum calcium. Reaching this goal often requires exogenous supplementation for female athletes, particularly those with lactose intolerance. The gut cannot absorb more than 500 mg of calcium at once, so calcium intake should be spread throughout the day to maximize absorption [125]. Vitamin D improves intestinal absorption of calcium (as well as renal reabsorption and bone release), so adequate vitamin D levels are needed to achieve adequate calcium. Most calcium supplements contain vitamin D and many dairy products are fortified with vitamin D.

Vitamin D is another micronutrient important for maintaining bone health, skeletal muscle health, immunity, and injury prevention [15]. In the past decade, vitamin D has been a fertile research topic for exercise physiologists and consensus on optimal vitamin D levels, supplementation, and overall function has yet to be reached; a thorough summary of the various findings is outside the scope of this review. Vitamin D is a fat-soluble compound primarily synthesized through exposure to sunlight. Dietary availability of vitamin D is low [126]. Athletes who are at risk for low vitamin D include those living at northern/southern latitudes (> 35th parallel), athletes who train indoors, and those who aggressively cover their skin with sunscreen or clothing while outside [15, 127]. A study of 102 National Collegiate Athletic Association (NCAA) female athletes at a single institution found 21.5% to have abnormal vitamin D measures [128]; a separate study found 80% of female athletes and dancers to have abnormal vitamin D values [129]. The Institute of Medicine definition of vitamin D levels is shown in Table 3 [130]. It is reasonable for female athletes to aim to have 25-OH-vitamin D levels > 50 nM to protect their bones. This can be achieved with daily maintenance supplementation of 1000–2000 IU vitamin D3, depending on time of year and regular sun exposure.

**Table 3:** Institute of Medicine levels of vitamin D concentrations [130]

25-OH-vitamin D concentration (nM)	Vitamin D status
< 12.5	Very deficient
12.5– < 30	Deficient
30–50	Inadequate
> 50	Adequate
> 180	Toxic

### Hydration

A complete discussion of the hydration needs of female athletes is beyond the scope of this review. Briefly, female athletes can think of their hydration needs in two buckets: daily maintenance hydration and pre-, intra-, and post-exercise hydration. Recommendations specific for female athletes are lacking; thus, when interpreting data and guidelines from predominately male studies, female-specific physiology needs to be considered. Estrogen receptors and progesterone receptors are found in the hypothalamus, cardiovascular system, and kidney—organs all implicated in fluid balance and necessarily susceptible to cyclic changes in sex hormones [131]. These hormonal fluctuations, however, seem to have minimal effects on sodium and fluid handling, suggesting that the osmostat set point changes with the menstrual cycle to maintain fluid volume [131, 132].

During the luteal phase, under the influence of high levels of progesterone, basal body temperature can increase 0.5–1.0 °C [133]. Despite the increase in basal body temperature during the luteal phase, current evidence does not suggest an increased risk for heat illness (often secondary to dehydration) in women compared to men [134, 135]. Through a series of complex hormonal pathways, the increased levels of estrogen and progesterone during the luteal phase lead to increased fluid retention [136]. Perhaps paradoxically, intravascular volume can be depleted during the luteal phase due to extravasation [137].

Daily maintenance hydration is typically achieved without directed fluid plans or goals [15, 138]. In the United States, women are recommended to consume 2.7 L day^−1^ water, with 2.2 L consumed as liquids and the remainder coming from food [139]. This recommendation is likely sufficient for the basal needs of female athletes but may not meet the requirements for exercising athletes, especially in extreme environments. Methods for assessing hydration status are shown in Table 4 [138]. If an athlete is dehydrated prior to exercise, a slow rehydration process is preferred to rapid fluid intake [15]. This can be achieved by consuming 5–10 mL kg^−1^ water 2–4 h prior to exercise [15]. The proper hydration strategy during exercise has been a hotly debated topic over the past several decades. For most athletes in most situations, consuming 0.4–0.8 L h^−1^ is sufficient, and this can be achieved with a “drink to thirst” (i.e., drinking when thirsty) strategy without loss of performance [15, 138, 140]. Interindividual variability of sweat composition makes defining the ideal fluid composition to replenish lost electrolytes difficult [141]. Practically, athletes should try to consume 20–30 mmol L^−1^ sodium and 2–5 mmol L^−1^ potassium; this can be achieved by drinking water and supplementing with snacks or by drinking a sports beverage [138, 142]. After exercise, women should consume fluids at a modest rate in conjunction with sodium and potassium to make up any fluid deficit [143]. Similar to prehydration, rapid fluid consumption is not a recommended strategy immediately following exercise.

**Table 4:** Biomarkers of hydration status. Adapted from ACSM guidelines with permission [138]

Measure	Practicality	Validity (acute vs. chronic changes)	EUH cut-off
TBW	Low	Acute and chronic	< 2%
Plasma osmolality	Medium	Acute and chronic	< 290 mOsm
Urine specific gravity	High	Chronic	< 1.020 g mL^−1^
Urine osmolality	High	Chronic	< 700 mOsm
Body weight	High	Acute and chronic^a^	< 1%

*EUH* euhydration, *TBW* total body water

^a^Potentially confounded by changes in body composition during very prolonged assessment periods

There are two primary dangers for aberrant hydration with exercise: heat illness (heat exhaustion, heat stroke, hyperthermia) and hyponatremia. Women typically have lower metabolic rates during exercise and less body mass compared to men, leading to lower sweat rates and less water loss during exercise [138]. Dehydration is a significant risk factor for developing heat illnesses [138]. This risk is compounded by hot environments and lack of acclimatization. Readers are directed to the consensus statement for training and competing in the heat for specific recommendations on preventing and treating heat illness [144]. Exercise-associated hyponatremia (EAH) typically occurs when athletes consume free water far in excess of their losses or without concomitant electrolyte replenishment [145]. Risk factors for EAH include overdrinking during exercise, weight gain during exercise, exercise duration > 4 h, inexperience, inadequate training, slow pace, high or low BMI, and readily available fluids [145]. EAH is more common in women because they possess more of these listed risk factors; after accounting for BMI and exercise time, the sex difference is no longer observable [145]. When partaking in events lasting several hours, specific hydration plans may be beneficial for preventing EAH and maintaining performance [144]. Drinking to thirst is perhaps the safest strategy [145], but athletes may prefer specific plans if they feel they are at risk for over- or underhydration or for ease-of-mind, and they should work with a sports dietitian to develop these plans. Readers are further directed to the most recent EAH consensus statement [145] for more information.

## Conclusions

In this review, we have outlined the paucity of research specific to female athletes, described the medical sequelae of inadequate nutritional intake, provided a model to satisfy the nutritional needs of female athletes, and synthesized many recommendations about nutrition and hydration tailored to the female athlete. In brief, female athletes should aim for EA of approximately 45 kcal kg^−1^ FFM day^−1^ for optimal health and performance and maintenance of physique. Low EA can manifest as RED-S, a constellation of many symptoms of various physiologic systems that can be negatively affected by inadequate nutrition. Female athletes should pay close attention to their menstrual cycles to monitor for changes or irregularity that may signify nutritional deficiency and are reminded that missing cycles is not “normal for an athlete”. Female athletes are encouraged to eat a diverse range of foods to ensure adequate micronutrient intake. Micronutrients that are particularly important for female athletes include iron, calcium, and vitamin D. It is not unreasonable for female athletes to supplement their diets with 1000–2000 IU vitamin D pills daily. For most athletes, drinking when thirsty will provide adequate water intake and fluid balance. Female athletes are encouraged to consult sports dietitians to develop individualized nutrition plans and to be wary of advice pedaled by so-called “experts” on social media.

The banality of “more research is needed”—touted at the end of many original research articles and reviews—is no banality when it comes to women’s sport research. The many complexities of female physiology, including the menstrual cycle and body composition, have yet to be fully investigated for roles in health and performance. Well-controlled, carefully planned studies are needed to tease apart these complexities—this comes at great financial cost.

Since the passage of Title IX in the United States in 1972 (federal legislature preventing sex-based discrimination of funding in education programs), American women’s sports have seen marked growth in participation levels consisting of about 1000% increase in high school athletics and 600% increase in collegiate athletics [146]. Exercise physiology, performance, and nutrition research specific for women has lagged behind this explosion of participation. As such, women are subjected to potentially suboptimal recommendations from coaches, trainers, physicians, and others due to erroneous application of the results of studies of men. Few female professional sports associations have the power, money, or public reach that male sports associations have; consequently, there are few financial stakeholders in the success of female athletes to drive investment into research. The number of athletes who achieve professional success, however, is vanishingly low: investment should occur at all levels of age and ability equally across sexes to keep athletes on the field, having fun, performing at their peaks, and fostering a lifelong love of sport.

## References

[1] Costello JT, Bieuzen F, Bleakley CM (2014). Where are all the female participants in sports and exercise medicine research?. Eur J Sport Sci.

[2] Brookshire B. Women in sports are often underrepresented in science. 2016. https://www.sciencenews.org/blog/scicurious/women-sports-are-often-underrepresented-science. Cited 2020.

[3] Vescovi JD (2011). The menstrual cycle and anterior cruciate ligament injury risk: implications of menstrual cycle variability. Sports Med.

[4] Johnson ML, Pipes L, Veldhuis PP, Farhy LS, Boyd DG, Evans WS (2008). AutoDecon, a deconvolution algorithm for identification and characterization of luteinizing hormone secretory bursts: description and validation using synthetic data. Anal Biochem.

[5] Drinkwater BL, Nilson K, Chesnut CH, Bremner WJ, Shainholtz S, Southworth MB (1984). Bone mineral content of amenorrheic and eumenorrheic athletes. N Engl J Med.

[6] Yeager KK, Agostini R, Nattiv A, Drinkwater B (1993). The female athlete triad: disordered eating, amenorrhea, osteoporosis. Med Sci Sports Exerc.

[7] De Souza MJ, Williams NI, Nattiv A, Joy E, Misra M, Loucks AB (2014). Misunderstanding the female athlete triad: refuting the IOC consensus statement on relative energy deficiency in sport (RED-S). Br J Sports Med.

[8] Holtzman B, Ackerman KE (2019). Measurement, determinants, and implications of energy intake in athletes. Nutrients.

[9] Torstveit M, Sundgot-Borgen J (2005). Participation in leanness sports but not training volume is associated with menstrual dysfunction: a national survey of 1276 elite athletes and controls. Br J Sports Med.

[10] Kong P, Harris LM (2015). The sporting body: body image and eating disorder symptomatology among female athletes from leanness focused and nonleanness focused sports. J Psychol.

[11] Sundgot-Borgen J (1993). Prevalence of eating disorders in elite female athletes. Int J Sport Nutr.

[12] Bratland-Sanda S, Sundgot-Borgen J (2013). Eating disorders in athletes: overview of prevalence, risk factors and recommendations for prevention and treatment. Eur J Sport Sci.

[13] Ackerman KE, Holtzman B, Cooper KM, Flynn EF, Bruinvels G, Tenforde AS (2019). Low energy availability surrogates correlate with health and performance consequences of relative energy deficiency in sport. Br J Sports Med.

[14] Burke LM, Lundy B, Fahrenholtz IL, Melin AK (2018). Pitfalls of conducting and interpreting estimates of energy availability in free-living athletes. Int J Sport Nutr Exerc Metab.

[15] Thomas DT, Erdman KA, Burke LM (2016). Position of the Academy of Nutrition and Dietetics, Dietitians of Canada, and the American College of Sports Medicine: nutrition and athletic performance. J Acad Nutr Diet.

[16] Nattiv A, Loucks AB, Manore MM, Sanborn CF, Sundgot-Borgen J, Warren MP (2007). American College of Sports Medicine position stand. The female athlete triad. Med Sci Sports Exerc.

[17] Loucks AB (2004). Energy balance and body composition in sports and exercise. J Sports Sci.

[18] Loucks AB, Verdun M, Heath EM (1998). Low energy availability, not stress of exercise, alters LH pulsatility in exercising women. J Appl Physiol.

[19] Loucks AB (2013). Energy balance and energy availability. The encyclopaedia of sports medicine: an IOC medical commission publication.

[20] Lieberman JL, De Souza MJ, Wagstaff DA, Williams NI (2018). Menstrual disruption with exercise is not linked to an energy availability threshold. Med Sci Sports Exerc.

[21] Reed JL, De Souza MJ, Mallinson RJ, Scheid JL, Williams NI (2015). Energy availability discriminates clinical menstrual status in exercising women. J Int Soc Sports Nutr.

[22] Stellingwerff T (2018). Case study: body composition periodization in an olympic-level female middle-distance runner over a 9-year career. Int J Sport Nutr Exerc Metab.

[23] Mountjoy M, Sundgot-Borgen J, Burke L, Carter S, Constantini N, Lebrun C (2014). The IOC consensus statement: beyond the Female athlete triad-relative energy deficiency in sport (RED-S). Br J Sports Med.

[24] Mountjoy M, Sundgot-Borgen JK, Burke LM, Ackerman KE, Blauwet C, Constantini N (2018). IOC author consensus statement update 2018: relative energy deficiency in sport (RED-S). Br J Sports Med.

[25] Constantini NW. Medical concerns of the dancer. Book of Abstracts. In: FIMS World Congress of Sports Medicine Budapest; 2002. p 151.

[26] De Souza MJ, Nattiv A, Joy E, Misra M, Williams NI, Mallinson RJ (2014). 2014 female athlete triad coalition consensus statement on treatment and return to play of the female athlete triad: 1st international conference held in San Francisco, California, May 2012 and 2nd international conference held in Indianapolis, Indiana, May 2013. Br J Sports Med.

[27] Elliott-Sale KJ, Tenforde AS, Parziale AL, Holtzman B, Ackerman KE (2018). Endocrine effects of relative energy deficiency in sport. Int J Sport Nutr Exer Metab.

[28] Loucks AB, Thuma JR (2003). Luteinizing hormone pulsatility is disrupted at a threshold of energy availability in regularly menstruating women. J Clin Endocrinol Metab.

[29] Zanker CL, Swaine IL (1998). Relation between bone turnover, oestradiol, and energy balance in women distance runners. Br J Sports Med.

[30] Ihle R, Loucks AB (2004). Dose-response relationships between energy availability and bone turnover in young exercising women. J Bone Miner Res.

[31] Ackerman KE, Nazem T, Chapko D, Russell M, Mendes N, Taylor AP (2011). Bone microarchitecture is impaired in adolescent amenorrheic athletes compared with eumenorrheic athletes and nonathletic controls. J Clin Endocrinol Metab.

[32] Ackerman KE, Putman M, Guereca G, Taylor AP, Pierce L, Herzog DB (2012). Cortical microstructure and estimated bone strength in young amenorrheic athletes, eumenorrheic athletes and non-athletes. Bone.

[33] Ackerman KE, Cano-Sokoloff N, Denm G, Clarke HM, Lee H, Misra M (2015). Fractures in relation to menstrual status and bone parameters in young athletes. Med Sci Sports Exerc.

[34] Schorr M, Miller KK (2017). The endocrine manifestations of anorexia nervosa: mechanisms and management. Nat Rev Endocrinol.

[35] Rowland T (2012). Iron deficiency in athletes: an update. Am J Lifestyle Med.

[36] Sim M, Garvican-Lewis LA, Cox GR, Govus A, McKay AKA, Stellingwerff T (2019). Iron considerations for the athlete: a narrative review. Eur J Appl Physiol.

[37] Hess SY, Zimmermann MB, Arnold M, Langhans W, Hurrell RF (2002). Iron deficiency anemia reduces thyroid peroxidase activity in rats. J Nutr.

[38] Beard J, Tobin B, Green W (1989). Evidence for thyroid hormone deficiency in iron-deficient anemic rats. J Nutr.

[39] Petkus DL, Murray-Kolb LE, De Souza MJ (2017). The unexplored crossroads of the female athlete triad and iron deficiency: a narrative review. Sports Med.

[40] Spaulding-Barclay MA, Stern J, Mehler PS (2016). Cardiac changes in anorexia nervosa. Cardiol Young.

[41] Rickenlund A, Eriksson MJ, Schenck-Gustafsson K, Hirschberg AL (2005). Amenorrhea in female athletes is associated with endothelial dysfunction and unfavorable lipid profile. J Clin Endocrinol Metab.

[42] O'Donnell E, Scheid JL, West SL, De Souza MJ (2019). Impaired vascular function in exercising anovulatory premenopausal women is associated with low bone mineral density. Scand J Med Sci Sports.

[43] Kaiserauer S, Snyder AC, Sleeper M, Zierath J (1989). Nutritional, physiological, and menstrual status of distance runners. Med Sci Sports Exerc.

[44] Friday KE, Drinkwater BL, Bruemmer B, Chesnut C, Chait A (1993). Elevated plasma low-density lipoprotein and high-density lipoprotein cholesterol levels in amenorrheic athletes: effects of endogenous hormone status and nutrient intake. J Clin Endocrinol Metab.

[45] O'Donnell E, Goodman JM, Mak S, Murai H, Morris BL, Floras JS (2015). Discordant orthostatic reflex renin-angiotensin and sympathoneural responses in premenopausal exercising-hypoestrogenic women. Hypertension.

[46] Walsh NP (2019). Nutrition and athlete immune health: new perspectives on an old paradigm. Sports Med.

[47] Cohen S, Tyrrell DA, Smith AP (1991). Psychological stress and susceptibility to the common cold. N Engl J Med.

[48] Cohen S, Doyle WJ, Alper CM, Janicki-Deverts D, Turner RB (2009). Sleep habits and susceptibility to the common cold. Arch Intern Med.

[49] Schwellnus MP, Derman WE, Jordaan E, Page T, Lambert MI, Readhead C (2012). Elite athletes travelling to international destinations > 5 time zone differences from their home country have a 2-3-fold increased risk of illness. Br J Sports Med.

[50] Patel D (2011). Occupational travel. Occup Med (Lond).

[51] Melin A, Tornberg AB, Skouby S, Faber J, Ritz C, Sjodin A (2014). The LEAF questionnaire: a screening tool for the identification of female athletes at risk for the female athlete triad. Br J Sports Med.

[52] Drew M, Vlahovich N, Hughes D, Appaneal R, Burke LM, Lundy B (2018). Prevalence of illness, poor mental health and sleep quality and low energy availability prior to the 2016 Summer Olympic Games. Br J Sports Med.

[53] Drew MK, Vlahovich N, Hughes D, Appaneal R, Peterson K, Burke L (2017). A multifactorial evaluation of illness risk factors in athletes preparing for the Summer Olympic Games. J Sci Med Sport.

[54] Nova E, Samartin S, Gomez S, Morande G, Marcos A (2002). The adaptive response of the immune system to the particular malnutrition of eating disorders. Eur J Clin Nutr.

[55] Marcos A (1997). The immune system in eating disorders: an overview. Nutrition.

[56] Reas DL, Ro O (2017). Investigating the DSM-5 severity specifiers based on thinness for adults with anorexia nervosa. Int J Eat Disord.

[57] Heikura IA, Uusitalo ALT, Stellingwerff T, Bergland D, Mero AA, Burke LM (2018). Low energy availability is difficult to assess but outcomes have large impact on bone injury rates in elite distance athletes. Int J Sport Nutr Exerc Metab.

[58] Woodward B (1998). Protein, calories, and immune defenses. Nutr Rev.

[59] Nova E, Varela P, Lopez-Vidriero I, Toro O, Cenal MJ, Casas J (2001). A one-year follow-up study in anorexia nervosa. Dietary pattern and anthropometrical evolution. Eur J Clin Nutr.

[60] Field CJ, Gougeon R, Marliss EB (1991). Changes in circulating leukocytes and mitogen responses during very-low-energy all-protein reducing diets. Am J Clin Nutr.

[61] Melin A, Tornberg ÅB, Skouby S, Møller SS, Sundgot-Borgen J, Faber J (2015). Energy availability and the female athlete triad in elite endurance athletes. Scand J Med Sci Sports.

[62] Melin ATÅB, Skouby S, Faber J, Ritz C, Sjödin A, Sundgot-Borgen J (2014). The LEAF questionnaire: a screening tool for the identification of female athletes at risk for the female athlete triad. Br J Sports Med.

[63] Meng K, Qiu J, Benardot D, Carr A, Yi L, Wang J (2020). The risk of low energy availability in Chinese elite and recreational female aesthetic sports athletes. J Int Soc Sports Nutr.

[64] Whitney KE, Holtzman B, Parziale A, Ackerman KE (2019). Urinary incontinence is more common in adolescent female athletes with low energy availability. Orthop J Sports Med.

[65] Reardon CL, Hainline B, Aron CM, Baron D, Baum AL, Bindra A (2019). Mental health in elite athletes: International Olympic Committee consensus statement (2019). Br J Sports Med.

[66] Meehan KG, Loeb KL, Roberto CA, Attia E (2006). Mood change during weight restoration in patients with anorexia nervosa. Int J Eat Disord.

[67] Attia E, Walsh BT (2009). Behavioral management for anorexia nervosa. N Engl J Med.

[68] Green MW, Elliman NA, Wakeling A, Rogers PJ (1996). Cognitive functioning, weight change and therapy in anorexia nervosa. J Psychiatr Res.

[69] Vanheest JL, Rodgers CD, Mahoney CE, De Souza MJ (2014). Ovarian suppression impairs sport performance in junior elite female swimmers. Med Sci Sports Exerc.

[70] Woods AL, Garvican-Lewis LA, Lundy B, Rice AJ, Thompson KG (2017). New approaches to determine fatigue in elite athletes during intensified training: Resting metabolic rate and pacing profile. PLoS ONE.

[71] Baltgalvis KA, Greising SM, Warren GL, Lowe DA (2010). Estrogen regulates estrogen receptors and antioxidant gene expression in mouse skeletal muscle. PLoS ONE.

[72] Lowe DA, Baltgalvis KA, Greising SM (2010). Mechanisms behind estrogen's beneficial effect on muscle strength in females. Exerc Sport Sci Rev.

[73] Oosthuyse T, Bosch AN (2012). Oestrogen's regulation of fat metabolism during exercise and gender specific effects. Curr Opin Pharmacol.

[74] Devries MC, Hamadeh MJ, Phillips SM, Tarnopolsky MA (2006). Menstrual cycle phase and sex influence muscle glycogen utilization and glucose turnover during moderate-intensity endurance exercise. Am J Physiol Regul Integr Comp Physiol.

[75] Wallis GA, Dawson R, Achten J, Webber J, Jeukendrup AE (2006). Metabolic response to carbohydrate ingestion during exercise in males and females. Am J Physiol Endocrinol Metab.

[76] McNulty KL, Elliott-Sale KJ, Dolan E, Swinton PA, Ansdell P, Goodall S (2020). The effects of menstrual cycle phase on exercise performance in eumenorrheic women: a systematic review and meta-analysis. Sports Med.

[77] Houltham SD, Rowlands DS (2014). A snapshot of nitrogen balance in endurance-trained women. Appl Physiol Nutr Metab.

[78] Lariviere F, Moussalli R, Garrel D (1994). Increased leucine flux and leucine oxidation during the luteal phase of the menstrual cycle in women. Am J Physiol Endocrinol Metab.

[79] Kriengsinyos W, Wykes LJ, Goonewardene LA, Ball RO, Pencharz PB (2004). Phase of menstrual cycle affects lysine requirement in healthy women. Am J Physiol Endocrinol Metab.

[80] Lamont L, Lemon P, Bruot B (1987). Menstrual cycle and exercise effects on protein catabolism. Med Sci Sports Exerc.

[81] Bailey SP, Zacher CM, Mittleman KD (2000). Effect of menstrual cycle phase on carbohydrate supplementation during prolonged exercise to fatigue. J Appl Physiol.

[82] Rehrer NJ, McLay-Cooke RT, Sims ST (2017). Nutritional strategies and sex hormone interactions in women. Sex hormones exercise and women.

[83] Spriet LL (2014). New insights into the interaction of carbohydrate and fat metabolism during exercise. Sports Med.

[84] Cermak NM, van Loon LJ (2013). The use of carbohydrates during exercise as an ergogenic aid. Sports Med.

[85] Campbell SE, Angus DJ, Febbraio MA (2001). Glucose kinetics and exercise performance during phases of the menstrual cycle: effect of glucose ingestion. Am J Physiol Endocrinol Metab.

[86] Zderic TW, Coggan AR, Ruby BC (2001). Glucose kinetics and substrate oxidation during exercise in the follicular and luteal phases. J Appl Physiol.

[87] Jeukendrup AE (2017). Training the gut for athletes. Sports Med.

[88] Kerksick CM, Arent S, Schoenfeld BJ, Stout JR, Campbell B, Wilborn CD (2017). International society of sports nutrition position stand: nutrient timing. J Int Soc Sports Nutr.

[89] Tarnopolsky MA (2000). Gender differences in metabolism; nutrition and supplements. J Sci Med Sport.

[90] Burke LM, Hawley JA, Wong SH, Jeukendrup AE (2011). Carbohydrates for training and competition. J Sports Sci.

[91] Jeukendrup AE, Jentjens RL, Moseley L (2005). Nutritional considerations in triathlon. Sports Med.

[92] Paul DR, Mulroy SM, Horner JA, Jacobs KA, Lamb DR (2001). Carbohydrate-loading during the follicular phase of the menstrual cycle: effects on muscle glycogen and exercise performance. Int J Sport Nutr Exerc Metab.

[93] Tarnopolsky MA, Zawada C, Richmond LB, Carter S, Shearer J, Graham T (2001). Gender differences in carbohydrate loading are related to energy intake. J Appl Physiol.

[94] McLay RT, Thomson CD, Williams SM, Rehrer NJ (2007). Carbohydrate loading and female endurance athletes: effect of menstrual-cycle phase. Int J Sport Nutr Exerc Metab.

[95] Walker JL, Heigenhauser GJ, Hultman E, Spriet LL (2000). Dietary carbohydrate, muscle glycogen content, and endurance performance in well-trained women. J Appl Physiol.

[96] James AP, Lorraine M, Cullen D, Goodman C, Dawson B, Palmer TN (2001). Muscle glycogen supercompensation: absence of a gender-related difference. Eur J Appl Physiol.

[97] Burke LM (2020). Ketogenic low-CHO, high-fat diet: the future of elite endurance sport?. J Physiol.

[98] Manore MM (2002). Dietary recommendations and athletic menstrual dysfunction. Sports Med.

[99] Leddy J, Horvath P, Rowland J, Pendergast D (1997). Effect of a high or a low fat diet on cardiovascular risk factors in male and female runners. Med Sci Sports Exerc.

[100] Horvath PJ, Eagen CK, Ryer-Calvin SD, Pendergast DR (2000). The effects of varying dietary fat on the nutrient intake in male and female runners. J Am Coll Nutr.

[101] Gerlach KE, Burton HW, Dorn JM, Leddy JJ, Horvath PJ (2008). Fat intake and injury in female runners. J Int Soc Sports Nutr.

[102] Fleming J, Sharman MJ, Avery NG, Love DM, Gómez AL, Scheett TP (2003). Endurance capacity and high-intensity exercise performance responses to a high-fat diet. Int J Sport Nutr Exerc Metab.

[103] Watkins BA, Li Y, Seifert MF (2001). Nutraceutical fatty acids as biochemical and molecular modulators of skeletal biology. J Am Coll Nutr.

[104] Albertazzi P, Coupland K (2002). Polyunsaturated fatty acids. Is there a role in postmenopausal osteoporosis prevention?. Maturitas.

[105] (IOM) IoM (2005). Dietary reference intakes for energy, carbohydrate, fiber, fat, fatty acids, cholesterol, protein, and amino acids.

[106] Fallon KE (2008). Screening for haematological and iron-related abnormalities in elite athletes-analysis of 576 cases. J Sci Med Sport.

[107] Malczewska J, Szczepanska B, Stupnicki R, Sendecki W (2001). The assessment of frequency of iron deficiency in athletes from the transferrin receptor-ferritin index. Int J Sport Nutr Exerc Metab.

[108] Parks RB, Hetzel SJ, Brooks MA (2017). Iron deficiency and anemia among collegiate athletes: a retrospective chart review. Med Sci Sports Exerc.

[109] Koehler K, Braun H, Achtzehn S, Hildebrand U, Predel HG, Mester J (2012). Iron status in elite young athletes: gender-dependent influences of diet and exercise. Eur J Appl Physiol.

[110] Tan D, Dawson B, Peeling P (2012). Hemolytic effects of a football-specific training session in elite female players. Int J Sports Physiol Perform.

[111] World Health Organization. Haemoglobin concentrations for the diagnosis of anaemia and assessment of severity. 2011 [cited Vitamin and Mineral Nutrition Information System; Available from: https://www.who.int/vmnis/indicators/haemoglobin.pdf.

[112] Peeling P, Blee T, Goodman C, Dawson B, Claydon G, Beilby J (2007). Effect of iron injections on aerobic-exercise performance of iron-depleted female athletes. Int J Sport Nutr Exerc Metab.

[113] Castell LM, Nieman DC, Bermon S, Peeling P (2019). Exercise-induced illness and inflammation: can immunonutrition and iron help?. Int J Sport Nutr Exerc Metab.

[114] Smith JA (1995). Exercise, training and red blood cell turnover. Sports Med.

[115] Pedlar CR, Brugnara C, Bruinvels G, Burden R (2018). Iron balance and iron supplementation for the female athlete: a practical approach. Eur J Sport Sci.

[116] Portal S, Epstein M, Dubnov G (2003). Iron deficiency and anemia in female athletes-causes and risks. Harefuah.

[117] Risser WL, Lee EJ, Poindexter HB, West MS, Pivarnik JM, Risser JM (1988). Iron deficiency in female athletes: its prevalence and impact on performance. Med Sci Sports Exerc.

[118] Beard J, Tobin B (2000). Iron status and exercise. Am J Clin Nutr.

[119] Saunders AV, Craig WJ, Baines SK, Posen JS (2013). Iron and vegetarian diets. Med J Aust.

[120] USDA. Dietary Guidelines for Americans. In: Agriculture USDo, editor 2015.

[121] Committee on Mineral Requirements for Cognitive and Physical Performance of Military Personnel, Committee on Military Nutrition Research, Food and Nutrition Board, Institute of Medicine (IOM). Mineral requirements for military personnel: levels needed for cognitive and physical performance during garrison training. Washington (DC): National Academies Press; 2006.

[122] Stoffel NU, Cercamondi CI, Brittenham G, Zeder C, Geurts-Moespot AJ, Swinkels DW (2017). Iron absorption from oral iron supplements given on consecutive versus alternate days and as single morning doses versus twice-daily split dosing in iron-depleted women: two open-label, randomised controlled trials. Lancet Haematol.

[123] Santiago P (2012). Ferrous versus ferric oral iron formulations for the treatment of iron deficiency: a clinical overview. Sci World J.

[124] McCormick R, Sim M, Dawson B, Peeling P (2020). Refining treatment strategies for iron deficient athletes. Sports Med.

[125] Harvey JA, Zobitz MM, Pak CY (1988). Dose dependency of calcium absorption: a comparison of calcium carbonate and calcium citrate. J Bone Miner Res.

[126] Bailey RL, Dodd KW, Goldman JA, Gahche JJ, Dwyer JT, Moshfegh AJ (2010). Estimation of total usual calcium and vitamin D intakes in the United States. J Nutr.

[127] Deldicque L, Francaux M (2015). Recommendations for healthy nutrition in female endurance runners: an update. Front Nutr.

[128] Villacis D, Yi A, Jahn R, Kephart CJ, Charlton T, Gamradt SC (2014). Prevalence of abnormal vitamin D levels among division I NCAA athletes. Sports Health.

[129] Constantini NW, Arieli R, Chodick G, Dubnov-Raz G (2010). High prevalence of vitamin D insufficiency in athletes and dancers. Clin J Sport Med.

[130] Del Valle HB, Yaktine AL, Taylor CL, Ross AC (2011). Dietary reference intakes for calcium and vitamin D.

[131] Stachenfeld NS (2008). Sex hormone effects on body fluid regulation. Exerc Sport Sci Rev.

[132] Maughan RJ, McArthur M, Shirreffs SM (1996). Influence of menstrual status on fluid replacement after exercise induced dehydration in healthy young women. Br J Sports Med.

[133] Casanova R, Chaung A, Goepfert AR, Hueppchen NA, Weiss PM, Beckmann CR (2019). Beckmann and Ling’s obstetrics and gynecology.

[134] Gifford RM, Todisco T, Stacey M, Fujisawa T, Allerhand M, Woods DR (2019). Risk of heat illness in men and women: a systematic review and meta-analysis. Environ Res.

[135] Marsh SA, Jenkins DG (2002). Physiological responses to the menstrual cycle: implications for the development of heat illness in female athletes. Sports Med.

[136] Giersch GEW, Charkoudian N, Stearns RL, Casa DJ (2020). Fluid balance and hydration considerations for women: review and future directions. Sports Med.

[137] Oian P, Tollan A, Fadnes HO, Noddeland H, Maltau JM (1987). Transcapillary fluid dynamics during the menstrual cycle. Am J Obstet Gynecol.

[138] Sawka MN, Burke LM, Eichner ER, Maughan RJ, Montain SJ, American College of Sports Medicine (2007). American College of Sports Medicine position stand. Exercise and fluid replacement. Med Sci Sports Exerc.

[139] Erdman J, Appel L (2005). Dietary reference intake for water, potassium, sodium, chloride, and sulfate.

[140] Dion T, Savoie FA, Asselin A, Gariepy C, Goulet ED (2013). Half-marathon running performance is not improved by a rate of fluid intake above that dictated by thirst sensation in trained distance runners. Eur J Appl Physiol.

[141] Baker LB, Jeukendrup AE (2011). Optimal composition of fluid-replacement beverages. Compr Physiol.

[142] Institute of Medicine (IOM) (1994). Fluid replacement and heat stress.

[143] Shirreffs SM, Sawka MN (2011). Fluid and electrolyte needs for training, competition, and recovery. J Sports Sci.

[144] Racinais S, Alonso JM, Coutts AJ, Flouris AD, Girard O, Gonzalez-Alonso J (2015). Consensus recommendations on training and competing in the heat. Br J Sports Med.

[145] Hew-Butler T, Rosner MH, Fowkes-Godek S, Dugas JP, Hoffman MD, Lewis DP (2015). Statement of the 3rd international exercise-associated hyponatremia consensus development conference, Carlsbad, California, 2015. Br J Sports Med.

[146] Brooke-Marciniak BA, de Varona D. Amazing things happen when you give female athletes the same funding as men. 2016: https://www.weforum.org/agenda/2016/08/sustaining-the-olympic-legacy-women-sports-and-public-policy/. Cited 3 Apr 2021.

